# From KDIGO 2012 towards KDIGO 2021 in idiopathic membranous nephropathy guidelines: what has changed over the last 10 years?

**DOI:** 10.1007/s40620-022-01493-9

**Published:** 2022-11-30

**Authors:** Stamatia Stai, Georgios Lioulios, Michalis Christodoulou, Aikaterini Papagianni, Maria Stangou

**Affiliations:** Department of Nephrology, Aristotle University of Thessaloniki, Hippokration Hospital, Thessaloniki, Greece

**Keywords:** Idiopathic membranous nephropathy (IMN), Antibodies against the M-type transmembrane phospholipase A2 receptor (anti-PLA2R), Alkylating agents, Calcineurin inhibitors (CNI), Rituximab

## Abstract

The recommendations in the Kidney Disease: Improving Global Outcomes (KDIGO) 2021 guidelines regarding Idiopathic Membranous Nephropathy (IMN) management include significant changes as compared to those published in 2012. According to the recent guidelines, a biopsy is not always needed for IMN diagnosis; since diagnosis can be allowed for by the detection of circulating antibodies against the M-type transmembrane phospholipase A2 receptor (anti-PLA2R). Moreover, alterations in anti-PLA2R concentrations, along with other serum and urinary markers, may guide further follow-up. The findings of numerous recent studies which compared different immunosuppressive treatments resulted in substantial changes in treatment indications in the KDIGO 2021 guidelines, suggesting the stratification of patients into four risk categories. The definition of resistant cases and relapses was likewise modified. All the above will lead to a more granular and personalized approach, whose results need to be tested over time. In this commentary, we discuss the changes in the 2012 and 2021 guidelines, adding information from the most recent literature.

**Graphical abstract:**

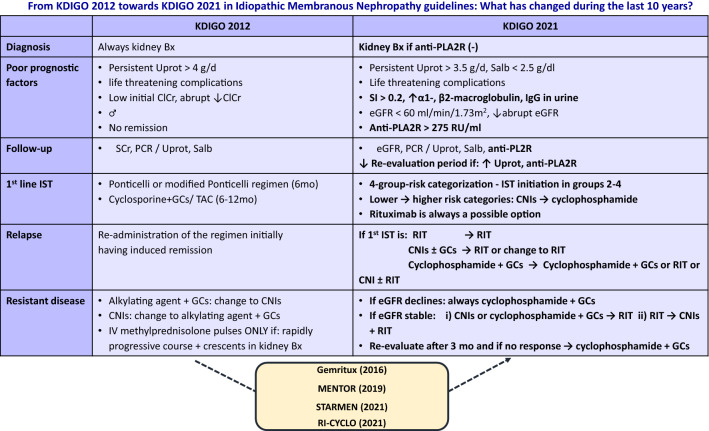

## Introduction

Membranous nephropathy (MN) is a common cause of nephrotic syndrome in adults, with the majority of patients being diagnosed between 30 and 50 years of age [1]. Approximately 75–80% of the cases are classified as idiopathic (IMN), since no association with another disease, such as lupus, or neoplasia, was found [1, 2]. The disease was first described by Heymann in 1959, author of a rat model of MN [3]. The discovery of antibodies directed against M-type transmembrane phospholipase A2 receptor (PLA2R), followed by other target molecules, including thrombospondin type-1 domain-containing 7A (THSD7A), neural epidermal growth factor-like 1 protein (NELL-1), semaphorin (SEMA) 3B, superoxide dismutase 2 (SOD2) and aldose reductase (AR) modulated the approach to the disease, leading to the development of follow-up and therapeutic strategies [1, 4]. In the Kidney Disease: Improving Global Outcomes (KDIGO) 2012 guidelines, alkylating agents are proposed as first-line treatment for patients fulfilling the criteria for immunosuppressive treatment administration, and calcineurin inhibitors (CNIs) were advised if the aforementioned drugs were contraindicated [5]. Following the 2012 guideline publication, several studies comparing the efficacy and safety of various regimens were conducted, leading to a substantial shift towards a personalized approach; many of these hints were included in the KDIGO 2021 guidelines.

## Overview of KDIGO 2012 guidelines for IMN

According to KDIGO 2012 guidelines, a kidney biopsy was necessary for diagnosing IMN.

Supportive treatment, based on renin–angiotensin–aldosterone system inhibitors (RAASis), treatment of dyslipidemia, prevention of thrombosis in high risk patients, combined with a balanced diet and strict arterial pressure control [6], were advised in order to reduce proteinuria and increase the chance of spontaneous remission.

Since all immunosuppressive regimens are potentially toxic, a period of 6 months of maximal support treatment was indicated, in order not to treat cases that would undergo spontaneous remission. Immunosuppressive treatment was recommended only in patients at high risk of progression, and specifically those with nephrotic syndrome and at least one of the following:

1. Proteinuria greater than 4 g per day, remaining at over 50% of baseline value, and not improving during 6 months of supportive treatment.
2. Severe and life-threatening complications of the nephrotic syndrome, such as thromboembolic episodes and anasarca.
3. An increase of at least 30% in serum creatinine (SCr) within 6–12 months from diagnosis, attributed to disease progression, combined with an estimated glomerular filtration rate (eGFR) at or below 25–30 ml/min/1.73 m^2^ [5].

Conversely, immunosuppressive treatment was not advised in patients with impaired kidney function (SCr > 3.5 g/dl or eGFR < 30 ml/min/1.73 m^2^) and imaging suggesting advanced chronic kidney disease (CKD). Even though the exact “point of no return” was not clearly defined, the above findings, combined with histological evidence of extensive interstitial fibrosis, tubular atrophy and glomerular sclerosis (according to some authors involving > 50% of the glomeruli), were considered to significantly reduce response probability, making the risks of immunoosuppressive drugs unacceptable [5, 6]. In this regard it is important to consider the regimens proposed for IMN treatment: the pharmacokinetics of cyclophosphamide, the alkylating agent of preference, is altered in patients with reduced eGFR, requiring dose adjustment [5, 7, 8], while CNIs are nephrotoxic [9, 10].

Definition of total remission included reduction of proteinturia to < 300 mg/day, combined with stable kidney function and serum albumin levels. Partial remission was defined as urinary protein excretion < 3.5 g/day and ≥ 50% reduction compared to initial values, combined with stable kidney function and normal or improved serum albumin levels [5].

The first choice was a a combination of alkylating agents (cyclophosphamide or chlorambucil) with corticosteroids [5]. Cyclophosphamide was preferred over chlorambucilbecause of fewer side effects (lower risk of myelosuppression and infectious complications), while the advantages on long-lasting remissions were described only in some low-quality studies [5, 11].

Regarding alkylating agents, their lower toxicity in intermittent administration in combination with corticosteroids (Ponticelli and modified Ponticelli regimen for chlorambucil and cyclophosphamide respectively) [5, 12, 13] for a duration of 6 months, was underlined.

Cyclosporine or tacrolimus were recommended as second-line treatment, mainly in case of side effects. Rituximab was not advised as first-line therapy, due to the lack of randomized controlled trials (RCTs) before the publication of the guidelines [5]. The recommended doses were 3.5–5 mg/kg/day orally for cyclosporine along with 0.15 mg/kg/day of prednisone for 6 months and 0.05–0.075 mg/kg/day for tacrolimus with or without prednisone, for 6–12 months. In both cases the drug should be divided into two doses, 12 h apart. If remission is achieved and maintained, tapering can be attempted every 4–8 weeks, to about 50% of the initial dose. Drug blood levels should be monitored regularly during the first period of treatment, and in case of unexplained ≥ 20% rise in serum creatinine [5].

A switch between the two calcinueurin inhibitors was advised if remission could not be achieved over 6 months [5]. Later, it was proposed that a > 30% rise in serum creatinine and/or persistent nephrotic-range proteinuria indicated non-responsiveness and treatment failure [6]; other reasons entailing the need for treatment change were rapid deterioration of the kidney function, or the presence of severe/life-threatening complications of the nephrotic syndrome [5]. In case of relapse, patients should receive the drug which previously achieved remission, although the cyclical alkylating agent/corticosteroid regimen should be repeated only once in patients who received it as first-line therapy [5].

## Brief comparison of KDIGO 2021 and 2012 IMN guidelines

In the following years, several new studies led to the guideline changes in 2021, concerning diagnostic approach, evaluation during follow up and treatment.

### Diagnosis of IMN

First of all, a biopsy is no longer required for IMN diagnosis [14]. Since anti-PLA2R antibodies, which are present in the serum of about 70% of patients, have a specificity for IMN of almost 100% and their titers correlate with the clinical status [1, 15], if the patient has the typical manifestations of nephrotic syndrome, along with positive antibodies, histological confirmation of the diagnosis is no longer needed. However, patients with anti-PLA2R antibodies should undergo a biopsy if they have an unusual immune profile [e.g., positive anti-nuclear antibodies (ANA)], in case of lack of response to treatment, if the nephrotic syndrome persists even though anti-PLA2R antibodies are negative, or if they have rapidly declining kidney function [14]. A study that significantly contributed to this modification included 838 patients (later expanded to 1152 participants) that underwent testing for anti-PLA2R between 2015 and 2018. All the patients with detectable anti-PLA2R antibodies had typical histological findings of MN. Moreover, in patients with preserved kidney function and no evidence of secondary causes, the biopsy did not provide information that altered their management [15].

### Evaluation of IMN activity—prognostic indices

A further novelty of the 2021 guidelines is the evaluation of disease activity and therapeutic choice, which is mainly based on the stratification of patients into four risk groups (Table 1).

**Table 1:** Stratification of patients into 3 risk groups (based on Toronto risk scale) and 4 risk groups (KDIGO 2021)

		Low risk	Moderate risk	High risk	Very high risk
Toronto risk score	eGFR (ml/min/1.73 m^2^)	Normal	Normal	Declining	–
	Uprot (gr/24 h)	< 4	4–8	> 8	–
KDIGO 2021	eGFR (ml/min/1.73 m^2^)	> 60	> 60	< 60	Rapid decline
	Salb	Normal	Normal	Low	Low
	Uprot (gr/24 h)	< 3.5	> 3.5	> 3.5	> 3.5
	Proteinuria selectivity index	< 0.2	< 0.2	> 0.2	> 0.2
	Serum anti-PLA2R (RU/ml)	< 50	< 50	> 50	> 50
	Urinary α1-microglobulin (μg/min)	< 40	< 40	> 40	> 40
	Urinary β2-microglobulin (mg/d)	< 250	< 250	> 250	> 250
	Urinary IgG (μg/min)	< 1	< 1	> 1	> 1
	Severe complications	No	No	No	Yes

*anti-PLA2R* anti-phospholipase A2 receptor antibodies; *eGFR* estimated glomerular filtration rate; *IgG* immunoglobin G; *N/A* not applicable; *Salb* serum albumin; *SI* selectivity index; *Uprot* proteinuria

The new score is based on eGFR, serum albumin, urinary protein excretion, proteinuria selectivity index, serum anti-PLA2R concentration, urinary levels of specific proteins (α1-microglobulin, β2-microglobulin, IgG) and the presence of severe and potentially life-threatening nephrotic syndrome complications.

This score is a step forward with respect to the previously described Toronto risk score (calculated as *e*^*x*^/(1 + *e*^*x*^), where *x* = 1.26 + 0.3 · persistent proteinuria − 0.3 · slope creatinine clearance − 0.05 · initial creatinine clearance), since its calculation requires a follow up of at least 6 months, unlike the newly proposed score [6].

Most of the parameters included in the 2021 risk stratification differ in KDIGO 2021 compared to 2012 guidelines, where only renal function and degree of proteinuria were considered. Several studies have highlighted the importance of urinary levels of α1, β1-microglobulin (both potential indicators of reduced tubular reabsorption capacity and glomerulonephritis progression) and IgG (marker of glomerular basement membrane damage, increased permeability and disrupted selectivity), as independent factors to estimate disease activity, decide treatment and follow the clinical response [16–18].

### Therapeutic strategy

KDIGO 2012 indications for the initiation of immunosuppressive treatment are summarized in Table 2; they include persistent proteinuria > 4 g/24 h, rapidly declining renal function or urinary excretion of low molecular weight proteins. Consequently, treatment is not advised in patients who have normal kidney function, along with urinary protein < 3.5 g/day and serum albumin > 3 g/dl or proteinuria reduction of > 50% after 6 months on RAASi [14].

**Table 2:** Brief presentation of the most important studies in the treatment of IMN

Author, year	Type of study	No. pts	Compared treatments	Main results	Side effects
Xie et al., 2012 [40]	Meta-analysis of 17 RCTs	696	Alkylating agents, CNI, MMF	Better effect of CNIs in CR rate in short term follow up	Cyclophosphamide: abnormal liver function, CNIs: hypertrichosis, hyperglycemia, MMF: Gastrointestinal complications
Chen et al., 2013 [41]	Meta-analysis of 36 RCTs	1762	Alkylating agents, CNI, MMF	IST: reduction of mortality and ESRD risk, increase of TR and CR	More frequent adverse events in Alkylating agents
Qiu et al., 2017 [22]	Meta-analysis of 21 RCTs	1187	Cyclophosphamide compared to CNIs	6 mo: Beneficial effect of CNIs; 12 mo: No difference between CNIs vs. cyclophosphamide	Cyclophosphamide: leukopenia, alopecia, liver damage, CNIs: hypertension, hyperuricemia, hirsutism, gingival hyperplasia
Ren et al., 2017 [30]	Network meta-analysis of 36 RCTs	2018	IST (Cyclophosphamide, CNIs, MMF, AZA) compared to RAASi	Cyclophosphamide: significantly reduced mortality or ESRD compared to control Cyclophosphamide; CNIs: significantly increased CR or PR; Cyclophosphamide better than CNIs in CR and PR	Cyclophosphamide: greater withdrawal rates due to side effects
Zheng et al., 2019 [42]	Network meta-analysis of 48 RCTs	2736	IST (Cyclophosphamide, CNIs, MMF, AZA) compared to non-IST	IST: increased probability of TR, reduced proteinuria	IST: Increased possibility of infections
Stangou et al., 2019 [35]	Retrospective study	491	Modified Ponticelli compared to CNIs + GCs	10 years: Clear superiority of cyclophosphamide in CR, PR, reduced relapse rate	Not estimated
Chen et al., 2010 [20]	RCT	73	Modified Ponticelli compared to CNIs (Tacrolimus) + GCs	6 mo: Superiority of Tacrolimus in CR rate; 12 mo: Similar outcome of two groups; 24 mo: Beneficial effects of Tacrolimus were reduced	Tacrolimus: Glucose intolerance, infections, elevated liver enzymes hypertension, transient renal impairment; Cyclophosphamide: Infections, elevated liver enzymes
Ramachandran et al., 2016 [43]	RCT	70	Modified Ponticelli compared to CNIs (Tacrolimus) + GCs	Comparable effects at 6 mo and 12 mo	Tacrolimus: Nephrotoxicity; Cyclophosphamide: Amenorrhea
Dahan et al., Gemritux study, 2016 [23]	RCT	75	Rituximab compared to RAASi	3 mo: beneficial effect of rituximab in anti-PLA2R	Comparable adverse events
Fervenza et al., MENTOR study, 2019 [25, 26]	RCT	130	Rituximab compared to CNIs	12 mo: Rituximab was noninferior to cyclosporine in CR or PR; 24 mo: Rituximab was superior to cyclosporine in maintaining proteinuria remission	Rituximab: Infusion-related reactions; CNIs: Increased serum creatinine levels, gastrointestinal events
Fernandez-Juarez et al., STARMEN study, 2021 [24]	RCT	86	sequential CNIs + rituximab compared to Modified Ponticelli	Significantly faster and sustained immunological and clinical remission in Modified Ponticelli group	More adverse events in Modified Ponticelli group: Leukopenia, infections
Scolari et al., RI-CYCLO, 2021 [27]	RCT	74	Rituximab compared to Modified Ponticelli	12 mo: increased probability of CR in Modified Ponticelli group, comparable results in the two groups regarding TR 24 mo: reduced probability of CR in the rituximab group, no difference between the two groups regarding TR	Rituximab: Infusion related reactions; Modified Ponticelli group: Leucopenia, pneumonia

*CNI* calcineurin inhibitor; *CR* complete remission; *GCs* glucocorticosteroids; *ESRD* end-stage renal disease; *IMN* idiopathic membranous nephropathy; *IST* immunosuppressive treatment; *MMF* mycophenolate mofetil; *mo* months; *PR* partial remission; *RAASi* renin–angiotensin–aldosterone system inhibitors; *RCT* randomized controlled trial; *TR* total remission (includes both CR and PR cases)

On the contrary, the 2021 KDIGO guidelines recommend treatment initiation in patients that are at least at moderate risk of kidney function impairment. Rituximab can be used in all three groups that require immunosuppressive treatment. Specifically, patients in the second risk group can receive symptomatic therapy, rituximab or calcineurin inhibitors. Even though administration of calcineurin inhibitors for a period of 6–12 months has been correlated with higher relapse rates, their use is not considered a “wrong tactic” in patients with normal eGFR and moderate risk for kidney function deterioration. The recommended use of calcineurin inhibitors is mainly based upon studies supporting a faster effect on proteinuria, compared to cyclophosphamide [19–22].

Furthermore, in the presence of negative prognostic factors, rituximab co-administration is possible, with the exception of patients without detectable anti-PLA2R antibodies after 6 months with a calcineurin-based treatment.

Consequently, in patients in the third group we can choose between rituximab, cyclophosphamide + glucocorticoids for 6 months and CNI + rituximab for at least 6 months. In the fourth risk group, cyclophosphamide along with glucocorticoids is still the treatment of choice, while rituximab can be an alternative, in the presence of contraindications. Here, it is important to point out that in the KDIGO 2021, calcineurin inhibitors are always co-administered with corticosteroids. Cyclophosphamide is the only alkylating agent proposed, while chlorambucil is no longer included in the algorithm due to the greater frequency of side effects [14].

Dose adjustments of the proposed regimens are summarized below:

1. Oral cyclophosphamide dose in the modified Ponticelli regimen has been increased from 2 to 2.5 mg/kg/day. For the first time two alternatives are suggested: the daily continuous scheme and the intravenous pulses. The drug dose is lower (1.5 mg/kg/day) in case of daily co-administration of 0.5 mg/kg/day of prednisone for a total of 6 months, over the cyclical scheme. Intravenous infusion of cyclophosphamide is advised in cases that should receive a lower cumulative dose (e.g., previous treatment with cyclophosphamide, women of reproductive age). In order to reduce the risk of malignancy, the total cyclophosphamide dose must not exceed 36 g, although for safety reasons the upper level has been set to 25 g for the general population and to 10 g for women planning to get pregnant.
2. Dose of cyclosporine has been re-defined to 3.5 mg/kg/day and of tacrolimus to 0.05–0.1 mg/kg/day, with their target trough levels ranging from 125 to 225 ng/ml and from 3 to 8 ng/ml, respectively. In both cases 10 mg/day of prednisone can be co-administered. Tapering can be attempted after 12 months, while in patients not having responded by month 4, treatment should be discontinued.
3. Rituximab can be either administered as two intravenous doses of 1 g with a 15-day interval between them, or as four doses of 375 mg/m^2^ given within 1 month (1 infusion per week) [14].

The main modifications in KDIGO 2021 guidelines are the inclusion of rituximab as a first line treatment and the exclusion of chlorambucil. The decision to upgrade rituximab was predictable after several randomized studies over the last decade compared it with other IMN treatments (Table 2). The Evaluate Rituximab Treatment for Idiopathic Membranous Nephropathy Study (GEMRITUX) was a multicenter RCT of 75 patients with severe resistant nephrotic syndrome after 6 months of therapy with maximal doses of RAASis. Thirty-eight of them continued receiving only RAASis for 6 more months, while the remaining 37 received two infusions (days 1 and 8) of 375 mg/m^2^ of rituximab. Although rituximab failed to achieve higher rates of proteinuria remission (complete or partial) by the end of the trial, anti-PLA2R concentrations were reduced by month 3 and serum albumin was increased at months 3 and 6, without safety concerns. A higher percentage of proteinuria remission was recorded in the post-RCT observational period [23]. Membranous nephropathy trial of rituximab (MENTOR) was another multicenter RCT, with 130 participants and a follow-up period of 24 months, that compared rituximab (two infusions of 1 g with a 15-day interval, re-administration of the same doses after 6 months if complete remission had not been achieved) with cyclosporine (12-month scheme). After 24 months, remission rates were higher in the rituximab arm, even though the difference was not statistically significant at one year. Moreover, cyclosporine discontinuation was followed by more IMN relapses [24–26]. Sequential treatment with tacrolimus and rituximab versus alternating corticosteroids and cyclophosphamide in IMN (STARMEN) was a randomized, open-label trial recruiting 86 patients with nephrotic syndrome that persisted after 6 months of follow-up. Half of the participants were treated with a 6-month cyclophosphamide/corticosteroid alternate regimen and half with tacrolimus combined with rituximab. The first scheme was more effective in achieving remission at 2 years and in immunological response induction (disappearance of anti-PLA2R antibodies) at months 3 and 6. This was attributed to the fact that cyclophosphamide, whose action is wider, provoked a more important decrease in circulating antibodies compared to the more “targeted” action of rituximab. Furthermore, remission in patients receiving the alkylating agent-based regimen was faster and, in most cases, complete, while in the majority of those treated with the CNI/rituximab combination, remission was partial. Nevertheless, it should be noted that rituximab administration when tacrolimus was being tapered increased complete remissions, and reduced the relapse rates as compared to after calcineurin inhibitor discontinuation [24]. Last but not least, there was the rituximab versus Steroids and Cyclophosphamide in the Treatment of Idiopathic Membranous Nephropathy (RI-CYCLO) trial, an open-label, pilot RCT with 74 IMN patients that exhibited proteinuria > 3.5 g/d, assigned either to rituximab infusions (1 g on days 1 and 15) or a cyclical cyclophosphamide/corticosteroid-based regimen (duration 6 months). Researchers found that rituximab did not differ in inducing complete remission and complete or partial remission at 12 and 24 months, orin the short-term safety profile. Moreover, rituximab was not more effective in reducing proteinuria, increasing serum albumin levels at 24 months and in decreasing anti-PLA2R concentrations, although the titers seemed to drop faster in the rituximab arm. Relapse rates were comparable among the two study arms [27].

Table 2 summarizes the results of the largest studies on treatment of IMN.

Alkylating agents may cause severe short- and long-term side effects, thus, their utilization should be limited to high-risk patients [14, 19, 27–29]. Moreover, chlorambucil has a less favorable adverse reaction profile compared to cyclophosphamide and patients with impaired renal function are more prone to their manifestation [11, 14, 30–32]. Its clinical use was reduced after the wide application of “the alternative Ponticelli regimen”, and the KDIGO 2021 guidelines formally exclude it from the IMN treatment choices.

### Refractory cases and relapse management

Even though re-emergence of proteinuria > 3.5 g/day in patients having achieved complete or partial remission is defined as a relapse, distinction between relapse and resistant IMN may be extremely difficult. The differential diagnosis can be facilitated by a combination of measurements including serum albumin, proteinuria and anti-PLA2R presence and titers. Fluctuations in antibody titers precede changes in the disease course and should be co-assessed with the clinical findings. Characteristically, the presence of anti-PLA2R antibodies is compatible with resistant disease. In case of relapse, rituximab, can be re-administered, while in patients on CNI ± prednisone, rituximab can be added to the CNI or therapy may be switched to rituximab, while in patients previously treated with cyclophosphamide + glucocorticoids, the scheme may be repeated once or the patient can be switched to one of the two alternatives. When the relapse occurs early in the disease course, further factors that might be responsible for treatment failure need to be investigated: these can include poor compliance, subtherapeutic drug levels, inefficient B-cell depletion and the presence of antibodies against rituximab [14].

Differences between KDIGO 2012 and 2021 guidelines are summarized in Tables 3 and 4.

**Table 3:** Overall approach to IMN—differences between KDIGO 2012 and 2021 [5, 14]

	KDIGO 2012	KDIGO 2021
Diagnosis	Kidney biopsy always required	Kidney biopsy required in anti-PLA2R (−)
Poor prognostic factors	Laboratory	
	1. Persistent (> 6 months of RAASi) Uprot > 4 g/d 2. Low initial ClCr 3. Abrupt deterioration of kidney function 4. Great negative slope of ClCr over 6 mo / > 30% elevation in SCr within 6–12 mo from diagnosis 5. Persistently elevated C5b-9* 6. Increased urinary excretion of LMW proteins (β2-microglobulin, IgG)*	1. Persistent (> 6 months of RAASi) Uprot > 3.5 g/d 2. Initial eGFR < 60 ml/min/1.73m^2^ 3. Abrupt deterioration of kidney function 4. Salb < 2.5 g/dl 5. Anti-PLA2R > 50 RU/ml** (especially if > 275 RU/ml) 6. Increased urinary excretion of LMW proteins (α1-microglobulin > 40 μg/min, β2-microglobulin > 250 mg/d, IgG > 1 μg/min) 7. SI > 0.2
	Clinical	
	1. Male gender 2. Severe/life- threatening NS complications 3. Persistent NS 4. Absence of remission 5. Hypertension*	1. Severe persistent NS 2. Life-threatening NS complications
	Histological	
	1. TA* 2. IF*	-
Follow-up	1. SCr 2. PCR/Uprot 3. Salb	1. eGFR 2. PCR/Uprot 3. Salb 4. Anti-PL2R (measured every 3–6 mo—shorter intervals in higher baseline values)

*anti-PLA2R* anti-phospholipase A2 receptor antibodies; *ClCr* creatinine clearance; *IF* interstitial fibrosis; *LMW* low molecular weight; *NS* nephrotic syndrome; *PCR* protein/creatinine ratio; *Uprot* proteinuria; *RAASi* renin–angiotensin–aldosterone system inhibitors; *Salb* serum albumin; *SCr* serum creatinine; *SI* selectivity index; *TA* tubular atrophy

^*^Low quality data; **cut-off values not validated

**Table 4:** Differences between KDIGO 2012 and 2021 IMN therapeutic strategies [5, 14]

	KDIGO 2012	KDIGO 2021
Conservative treatment	For ≤ 6 mo unless rapidly deteriorating kidney function or severe NS complications	For ≤ 6 mo (re-evaluation if high Uprot, anti-PLA2R or LMW), add IST if abrupt deterioration of kidney function or uncontrolled NS
First line IST	1. 6 mo of Ponticelli or modified Ponticelli regimen 2. 6mo of Cyclosporine + GCs or 6-12mo of Tacrolimus	1. Choice based on a 4-group-risk categorization 2. IST initiation advised in groups 2–4 3. RIT is always a possible option 4. Proceeding from lower to higher risk categories CNIs are replaced by cyclophosphamide 5. Cyclophosphamide-based regimen is first choice treatment in patients with worse expected outcome 6. Addition of GCs to CNIs is recommended over CNI monotherapy 7. Chlorambucil use is no longer advised 8. The possibility of IV cyclophosphamide infusion is examined
Relapse	Re-administration of the regimen initially having induced remission	1. If early relapse: search for factors responsible for previous treatment failure 2. If first IST is A. RIT: continue with RIT B. CNIs ± GCs: add RIT or change to RIT C. Cyclophosphamide + GCs: continue with cyclophosphamide + GCs or change to RIT or CNI ± RIT
Resistant disease	Definition: failure to achieve remission	Definition (none universally accepted): 1. In anti-PLA2R ( +) at high or stable concentrations after 1^st^ line IST even if “clinical remission” has been achieved 2. In anti-PLA2R (−): Persistent NS (> 6 mo) 3. Increase of Uprot after achieving levels of 2–3.5 g/d without Salb normalization 4. Small dense deposits in kidney biopsy (if diagnostic problem)
	1. Alkylating agent + GCs change to CNIs 2. CNI change to Alkylating Agent + GCs 3. IV methylprednisolone pulses ONLY if: rapidly progressive course interrupts steady evolution + crescents in kidney biopsy	1. Check compliance and efficacy 2. A. If eGFR declines: second treatment: always cyclophosphamide + GCs B. If eGFR stable: (i) CNIs or cyclophosphamide + GCs: change to RIT (ii) RIT change to: CNIs + RIT 3. Re-evaluate after 3 mo and if no response: switch to cyclophosphamide + GCs 4. Expert center (for bortezomib, anti-CD38, belimumab or higher doses of conventional IST)

*anti-PLA2R* anti-phospholipase A2 receptor antibodies; *CNIs* calcineurin inhibitors; *eGFR* estimated glomerular filtration rate; *GCs* glucocorticoids; *IST* immunosuppressive treatment; *LMW* low molecular weight; *NS* nephrotic syndrome; *RIT* rituximab; *Salb* serum albumin

^*^KDIGO 2012 advises not to use the alkylating agent-based regimen more than twice, while in the KDIGO 2021 the only relevant limitation has to do with exceeding the maximal tolerable cumulative dose

### Biomarkers during follow up

To some degree, decisions regarding treatment modifications are affected by anti-PLA2R titers, which should be measured every 3–6 months. Shorter re-assessment intervals are advised for patients with higher antibody concentrations at the beginning of follow-up. Values of < 2 RU/ml calculated with enzyme-linked immunosorbent assay (ELISA) indicate complete immunological remission; once achieved, if the patient was treated with rituximab or cyclophosphamide-glucocorticoid combination, immediate therapy discontinuation can be attempted, while if the patient was receiving CNIs (with or without corticosteroids), tapering should be gradual. In cases in which the anti-PLA2R concentrations remain over 50 RU/ml, if rituximab was the initial regimen, two additional infusions of 1 g (with a 15-day interval between them) are advised, while if the first choice was cyclophosphamide + glucocorticoids, the scheme should be replaced with rituximab, and if the initial treatment was CNIs + prednisone, CNI tapering and addition of either rituximab or cyclophosphamide + glucocorticoids is advised. Lastly, in case of antibody concentrations > 2 RU/ml and < 50 RU/ml, the approach is similar, with two modifications: (i) after cessation of the cyclophosphamide-glucocorticoid combination, the patient is simply monitored and (ii) CNI-based therapy is continued for 6 more months and then the patient is re-assessed [14].

## Further relevant issues

After the KDIGO 2012 guideline publication, several new studies were published.

In this context, the Toronto score emerged as an additional tool to assess the need forstarting immunosuppressive therapy. It can be calculated after 12–24 months of follow up, and combines SCr concentration, proteinuria levels and the change in eGFR, defining three risk categories (Table 1) [6, 33]. Administration of immunosuppressive drugs is advised in patients with high and moderate risk of kidney function impairment, while those in the mild risk group can continue with supportive therapy. Conditions that render the initiation of immunosuppressive agents urgent are proteinuria > 10 g/day or the inability to reduce it below 8 g/day after 3 months of conservative treatment, as well as an unexplained drop in eGFR [6].

The discovery of PLA2R in 2009 was of great importance in redesigning the approach to IMN [33]. The importance of anti-PLA2R is already pointed out in the KDIGO 2012 guidelines [5]. The detection of circulating anti-PLA2R and against THSD7A, as well as the identification of relevant antigens and immunoglobin in the glomerulus, is essential for the assessment of the immunological activity, modulating treatment decisions. If antibody tests are negative and PLA2R, THSD7A and other specific antibodies (e.g. NELL-1, SEMA 3B) are not found at the glomerular level after proper staining, and if the immunoglobins are mainly of IgG1-3 subclasses, a secondary MN may be suspected. In these patients antibodies against other podocyte antigens may be the cause. If circulating antibodies are not detectable, but the relevant IgG4 subtypes are detected in the kidney biopsy, IMN may be anti-PLA2R/anti-THSD7A-mediated, but inactive.

If a patient with immunologically active IMN is already on immunosuppressive treatment, high anti-PLA2R levels (without titer reduction) after 4–6 months are an indication to change regimen [6].

Changes in the treatment proposed in the KDIGO 2021 guidelines are also linked to the different safety profiles. The use of alkylating agents has been linked to better chances of preserving eGFR, thus preventing end stage renal disease, as well as lower mortality rates. Patients receiving CNIs display a higher risk of severe deterioration of kidney function [14, 19, 27–29] due to their time-dependent and non-reversible nephrotoxicity, related to increased transforming growth factor β (TGF-β) activity and characterized by the development of interstitial fibrosis and glomerulosclerosis [9, 10, 34, 35]. On the other hand, CNIs are more effective than alkylating agents in achieving rapid remission, while the difference is not significant in longer follow-up [19–22]. This advantage has been attributed to their additional antiproteinuric effects, not linked to their immunomodulatory actions, as they also reduce the levels of angiopoietin-like-4 protein in podocytes, thus promoting their selective repair. Besides, cyclosporine induces proteinuria reduction by directly targeting the cytoskeleton through stabilization of the actin-organizing protein synaptopodin and by inhibiting nuclear factor of activated T-cells in podocytes, as this factor induces apoptosis and glomerulosclerosis [21, 22, 36, 37]. The CNI-mediated restoration of the glomerular barrier integrity is a reversible effect, that recedes after drug discontinuation. This can justify to some extent the higher relapse rates among patients receiving CNIs compared to those treated with alkylating agents [22, 29, 35]

## Conclusion

The KDIGO 2021 guidelines are far more detailed and specific in comparison to those of 2012, overall adopting a personalized patient approach. A considerable number of changes have been proposed in several fields, including diagnosis and follow-up, with anti-PLA2R level measurement being central for both. Alkylating agents are no longer considered a first-line regimen for the majority of patients (although cyclophosphamide is still considered the best option for those with worse prognosis), while rituximab has become the preferred first-line treatment. CNI use is still advised, though limited to lower risk categories. Many issues are still not clarified. Recommendations for future research include exploration of novel antigens potentially pathogenic for IMN [38], as well as the definition of anti-PLA2R cut-off levels. Furthermore, the need to compare rituximab with cyclophosphamide, as well as an in-depth focus on cyclophosphamide in order to reduce its toxicity were highlighted. Finally, the efficacy and safety of novel anti-CD20 humanized monoclonal antibodies (such as ofatumumab, obinutuzumab, and ocrelizumab) in refractory and relapsing IMN and in patients who developed side effects of rituximab were added to the research agenda together with the need to study anti-Baff therapy (Belimumab), anti-plasma cell therapy, immunoadsorption, anti-complement therapy [39] and their monitoring.
